# The interactivity of sources and dietary levels of resistant starches – impact on growth performance, starch, and nutrient digestibility, digesta oligosaccharides profile, cecal microbial metabolites, and indicators of gut health in broiler chickens

**DOI:** 10.1016/j.psj.2024.104337

**Published:** 2024-09-24

**Authors:** Iyabo W. Oluseyifunmi, Jeferson Lourenco, Oluyinka A. Olukosi

**Affiliations:** ⁎ Department of Poultry Science, University of Georgia, Athens, GA 30602, USA; † Department of Animal and Dairy Science, University of Georgia, Athens, GA 30602, USA

**Keywords:** Resistant starch, growth performance, nutrient digestibility, gut morphometry, cecal short-chain fatty acid

## Abstract

In a 21-d study, 480 Cobb 500 (off-sex) male broiler chicks were used to investigate the effects of feeding different sources and levels of resistant starches (**RS**) on growth performance, nutrient and energy utilization, and intestinal health in broiler chickens. The birds were allocated to 10 dietary treatments in a 3 × 3 + 1 factorial arrangement. The factors were 3 RS-sources (**RSS**): banana starch (**BS**), raw potato starch (**RPS**), and high-amylose corn starch (**HCS**); each at 3 levels (**RSL**) 25, 50, or 100 g/kg plus a corn-soybean meal control diet. Birds and feed were weighed on d 0, 8, and 21. On d 21, samples of jejunal tissue and digesta were collected for chemical analysis. Data were analyzed using the mixed model procedure of JMP with factor levels nested with the control. In the 0 to 21 phase, the birds fed the RPS diets had higher (*P* = 0.011) FI than those fed HCS or control diets, and FCR was greater (*P* = 0.030) in birds that received BS diets than in other diets. RSS × RSL was significant (*P* < 0.05) for total tract nutrient retention, AME, and AMEn on d 21. The starch digestibility was higher (*P* < 0.001) in birds that received the control diet than in RS diets, and decreased as RS levels increased, except for HCS. The apparent metabolizable energy (**AME**) and nitrogen-corrected AME (**AMEn**) were higher (*P* < 0.001) in birds fed 100 g/kg HCS diet, with both decreasing with increasing levels of BS and RPS, except for HCS. Relative ileal oligosaccharides profile showed significant (*P* < 0.05) RSS × RSL with a higher relative abundance of Hex(3) (*P* = 0.01) and Pent(3) (*P* = 0.001) in HCS diets. In conclusion, RS may influence gut health and growth performance in broiler chickens through modulation of cecal SCFA and nutrient digestion, but these depend largely on the botanical origin and concentrations of individual RS.

## INTRODUCTION

Consumer concern coupled with the ban of antibiotic feed additives in some parts of the world have necessitated the search for feed components that could improve growth performance and maintain gut health in poultry (Vondruskova et al., 2010). Exogenous enzymes, probiotics, prebiotics, amino acids among others have been in use for modulating intestinal health but functional carbohydrates are another promising nutritional approach for improving intestinal health (Dobranowski and Stintzi, 2021). Resistant starches **(RS)** belong to the group of functional dietary fibers that are capable of modulating gut health, exhibiting prebiotic effects in the hindgut. As gut health is characterized by efficient digestion and nutrient absorption, the absence of gastrointestinal disorders, a balanced and stable intestinal microbiome, a robust immune system, and in all, an overall state of well-being (Bischoff, 2011)

Resistant starches are starches that escape digestion in the proximal intestinal tract and undergo fermentation in the ceca, producing beneficial metabolites such as short-chain fatty acids (**SCFA**) which are critical for gut health, enhanced immunity, and better nutrient utilization (Landon et al., 2012). There are 5 classes of RS based on sources and factors affecting their resistance to digestion (Champ, 2004). These include RS type 1 (**RS1**) which are starches that are encapsulated within plant cell walls and are inaccessible to amylolytic enzymes, present in unprocessed whole grains (Tan et al., 2021). RS type 2 (**RS2**) are naturally occurring RS granules and good examples are raw potato and green banana starches; RS type 3 (**RS3**) are starches that are retrograded and occurs when starchy foods are cooked and then cooled; RS type 4 (**RS4**) are chemically modified starches whereas the RS type 5 (**RS5**) are mainly starches that form complexes with lipids (Tan et al., 2021).

Resistant starches increase the proportion of fermentable carbohydrates relative to protein in the caeca, and the fermentable starches are possible prebiotics in the distal part of the digestive tract. The increased SCFA production lowers the caeca pH and creates a hostile environment for the harmful microbes preventing their proliferation, and this, in turn, dictates the rate and profile of SCFA production (Roy et al., 2006; Regassa and Nyachoti, 2018). The SCFA produced in the ceca include propionate, acetate and butyrate (Macfarlane and Macfarlane, 2003). Although acetate and propionate have beneficial impacts on health, butyrate seems most important for gut health and is reported as the SCFA most increased by RS consumption (Fuentes-Zaragoza et al., 2010; DeMartino and Cockburn, 2020). Dietary inclusion of 120 g/kg raw potato starch (**RPS**) for 35 d increased cecal acetate, propionate, and butyrate profile in Cherry Valley meat ducks (Qin et al., 2019). In a broiler study by Zhang et al. (2020), dietary levels of 40, 80, and 120 g/kg corn RS increased cecal concentrations of acetic and butyric acid. Fermentation of diets rich in RS and high amylose starch has also been reported to facilitate increased intraluminal SCFA concentrations and lowered digesta and fecal pH in pigs. (Tan et al., 2021).

Short-chain fatty acids support the healthy functioning of the lower gastrointestinal tract by influencing the metabolism of enterocytes and colonocytes, thereby affecting the intestinal musculature and vasculature. In mice and humans, RS consumption has been associated with various health benefits such as prebiotic, hypocholesterolemic, and hypoglycemic effects, improved gut barrier function, and reduced risk of colon cancer and inflammation (Bojarczuk et al., 2022). However, resistant starches belonging to the same group may behave differently based on the type of crystallinity exhibited and different botanical origins (Giuberti et al., 2015). The crystallinity pattern influences the degree of resistance to pancreatic amylase, which ultimately determines the digestibility of the starches and the oligosaccharide profile in the distal digestive tract. These dictate the degree of bacteria penetration into the starch granules and the rate of depolymerization, as well as availability for bacterial fermentation (Macfarlane and Macfarlane, 2003; Martinez-Puig et al., 2003).

In spite of the information available in the literature for effect of RS in different species, there is a dearth of information on how the site and extent of starch digestion, sources, and levels of resistant starches can interact to influence the growth performance and intestinal environment in broiler chickens. The aim of the current study was, therefore, to investigate how sources and levels of RS influence the growth performance and nutrient utilization in broiler chickens in the starter and grower phases. Possible effects on starch digestibility in the small intestine, gut morphology and morphometry, ileal oligosaccharides profile, cecal protein and SCFA profile, and jejunal expression of nutrient transporters, and gut integrity genes were used in accounting for the effect of the treatments on growth performance, nutrient and energy utilization by the birds.

## MATERIALS AND METHODS

### Birds and Housing

The Institutional Animal Care and Use Committee of the University of Georgia approved the experimental procedures (IACUC number: A2021-06-006).

### Animal Housing, Diets, and Experimental Design

The birds used in the experiment were raised in metabolism cages in a controlled environment following recommended lighting and temperature regimes for Cobb 500 broiler chickens. A total of 480 male broiler chicks (byproducts of the female line) were allocated to 10 treatments in a 3 × 3 + 1 nested factorial arrangement. There were eight replicates per treatment and 6 birds per replicate. The diets included a corn-soybean meal control diet and the factors, 3 RS sources (**RSS**): banana starch (**BS**), raw potato starch (**RPS**), and high-amylose corn starch (**HCS**); each at 3 inclusion levels (**RSL**) 25, 50, or 100 g/kg.

The analyzed chemical composition of the resistant starches is presented in Table 1. Feed and water were provided ad libitum throughout the experiment. The diets were presented as mash in both starter and grower phases and formulated to meet the nutrient and energy recommendations for Cobb 500 broiler chickens (Cobb-Vantress, 2018b). All the diets were isocaloric and isonitrogenous. The diet compositions are presented in Table 2, Table 3 for the starter and grower phases, respectively.

**Table 1:** Analyzed chemical profile of resistant starches used in the experiment (g/kg, as fed).

Resistant starch source	DM	Total starch	Resistant starch	CP	GE, kcal/kg
Banana starch	899	729	373	4.6	3,833
Raw potato starch	894	806	651	0.7	3,442
High-amylose corn starch	898	368	350	1.9	3,744

DM, dry matter; RS, resistant starch; GE, gross energy; CP, crude protein.

**Table 2:** Ingredients and Chemical Composition (g/kg) of the Starter Phase (d 0–8) diets.

Items	Control	Banana starch			Raw potato starch			Hi-amylose corn starch		
Corn	629	598	566	495	598	560	485	593	551	469
Banana starch		25	50	100						
Raw potato starch					25	50	100			
Hi-amylose corn starch								25	50	100
Soybean meal	325	328	330	340	328	335	348	329	336	350
Soybean oil	10	12	17	28	12	18	30	16	26	44
Dicalcium phosphate	19	19	19	19	19	19	19	19	19	19
Limestone	7.6	7.6	7.6	7.6	7.6	7.6	7.6	7.6	7.6	7.6
Sodium bicarbonate	2.0	2.0	2.0	2.0	2.0	2.0	2.0	2.0	2.0	2.0
Vitamin/TM premix	2.5	2.5	2.5	2.5	2.5	2.5	2.5	2.5	2.5	2.5
DL- Methionine	1.5	1.6	1.6	1.6	1.6	1.6	1.6	1.6	1.6	1.6
L- Lysine. HCl	1.1	1.3	1.3	1.3	1.3	1.3	1.3	1.3	1.3	1.3
Threonine	0.3	0.3	0.3	0.3	0.3	0.3	0.3	0.3	0.3	0.3
Salt	2.4	2.4	2.4	2.4	2.4	2.4	2.4	2.4	2.4	2.4
Total	1000	1000	1000	1000	1000	1000	1000	1000	1000	1000
Calculated nutrients, g/kg										
Protein, g/kg	212	211	210	211	211	211	211	211	211	212
ME, kcal/kg	2989	2974	2974	2975	2974	2975	2978	2975	2978	2974
Available P, g/kg	4.6	4.5	4.5	4.5	4.5	4.5	4.5	4.5	4.5	4.5
Ca, g/kg	9.0	9.0	9.0	9.0	9.0	9.0	9.1	9.0	9.0	9.1
Digestible amino acids, g/kg										
Lys	12.2	12.3	12.3	12.4	12.3	12.5	12.6	12.4	12.5	12.7
Met	4.8	4.9	4.8	4.8	4.9	4.9	4.8	4.9	4.8	4.8
Thr	8.3	8.2	8.2	8.2	8.2	8.3	8.3	8.2	8.2	8.3
TSAA	8.3	8.3	8.2	8.1	8.3	8.3	8.2	8.3	8.3	8.2
Analyzed nutrient composition, g/kg, as fed										
Dry matter	902	895	895	901	896	892	898	897	898	898
Protein	196	166	186	191	198	171	190	196	186	192
GE, kcal/kg	3984	3919	4197	4504	4657	4927	5246	3982	4039	4068
Total starch	380	416	399	374	382	379	385	388	376	342

The vitamin and mineral premix provided (per kg of diet): Vitamin A, 5,484 IU; vitamin D3, 2,643 IU; vitamin E, 11 IU; menadione sodium bisulfate, 4.38 mg; riboflavin, 5.49 mg, d-pantothenic acid, 11 mg; niacin, 44.1 mg, choline chloride, 771 mg; vitamin B12, 13.2 µg; biotin, 55.2 µg; thiamine mononitrate, 2.2 mg; folic acid, 990 µg; pyridoxine hydrochloride, 3.3 mg, I, 1.11 mg; Mn, 66.06 mg; Cu, 4.44 mg; Fe, 44.1 mg; Zn, 44.1 mg; Se, 300 µg.

**Table 3:** Ingredients and chemical composition (g/kg) of the grower phase (d8–21) diets.

Items	Control	Banana starch			Raw potato starch			Hi-amylose corn starch		
Corn	662	627	591	517	627	590	514	623	582	498
Banana starch		25	50	100						
Raw potato starch					25	50	100			
Hi-amylose corn starch								25	50	100
Soybean meal	287	292	297	310	292	298	312	292	299	314
Soybean oil	10.0	15.0	21.0	32.0	15.0	21.0	32.5	18.5	28.0	47.0
Dicalcium phosphate	18.2	18.3	18.4	18.4	18.4	18.5	18.8	18.4	18.5	18.8
Limestone	7.7	7.7	7.7	7.7	7.7	7.7	7.7	7.7	7.7	7.2
Sodium bicarbonate	2.0	2.0	2.0	2.0	2.0	2.0	2.0	2.0	2.0	2.0
Vitamin/trace minerals premix	2.5	2.5	2.5	2.5	2.5	2.5	2.5	2.5	2.5	2.5
DL- Methionine	1.5	1.6	1.6	1.6	1.6	1.6	1.6	1.6	1.6	1.6
L- Lysine.HCl	1.2	1.3	1.3	1.3	1.3	1.3	1.2	1.3	1.3	1.2
L-Threonine	0.1	0.2	0.2	0.2	0.2	0.2	0.2	0.2	0.2	0.2
Titanium dioxide	5.0	5.0	5.0	5.0	5.0	5.0	5.0	5.0	5.0	5.0
Salt	2.4	2.4	2.4	2.4	2.4	2.4	2.4	2.4	2.4	2.4
Total	1000	1000	1000	1000	1000	1000	1000	1000	1000	1000
Calculated nutrients, g/kg										
Protein	196	196	197	198	196	196	196	196	196	197
ME, kcal/kg	3010	3008	3010	3008	3007	3009	3007	3007	3007	3008
Available P, g/kg	4.35	4.36	4.37	4.35	4.37	4.37	4.39	4.36	4.36	4.38
Ca	8.7	8.8	8.8	8.8	8.8	8.8	8.9	8.8	8.8	8.8
Digestible amino acids, g/kg										
Lys	11.2	11.3	11.4	11.6	11.3	11.4	11.6	11.3	11.4	11.6
Met	4.6	4.7	4.7	4.6	4.7	4.7	4.6	4.7	4.7	4.6
Thr	7.5	7.5	7.5	7.6	7.5	7.5	7.6	7.5	7.5	7.6
TSAA	7.9	7.9	7.9	7.8	7.9	7.9	7.8	7.9	7.9	7.8
Analyzed nutrients, g/kg as fed										
Dry matter	903	904	906	911	907	903	916	912	911	914
Protein	180	176	179	174	182	180	188	181	181	194
Gross energy, kcal/kg	4335	4321	4385	4411	4361	4434	4382	4384	4409	4480
Total starch	439	452	425	416	459	441	428	418	428	423
Resistant starch	8.0	10.1	12.2	16.4	24.3	40.5	73.0	13.7	19.4	30.8

The vitamin and mineral premix provided (per kg of diet): Vitamin A, 5,484 IU; vitamin D3, 2,643 IU; vitamin E, 11 IU; menadione sodium bisulfate, 4.38 mg; riboflavin, 5.49 mg, d-pantothenic acid, 11 mg; niacin, 44.1 mg, choline chloride, 771 mg; vitamin B12, 13.2 µg; biotin, 55.2 µg; thiamine mononitrate, 2.2 mg; folic acid, 990 µg; pyridoxine hydrochloride, 3.3 mg, I, 1.11 mg; Mn, 66.06 mg; Cu, 4.44 mg; Fe, 44.1 mg; Zn, 44.1 mg; Se, 300 µg.

### Growth Performance Measurements

Birds and feed were weighed on d 0, 8, and 21 to evaluate growth performance response. Body weight gain (**WG**), feed intake (**FI**), and FCR were corrected for mortality.

### Nutrient Digestibility, Ileal Oligosaccharides, Cecal Short-Chain Fatty Acids, and Cecal Protein Measurements

On d 21, birds were euthanized by CO_2_ asphyxiation, and jejunal and ileal digesta, cecal content, and excreta were collected for digestibility measurement, the determination of the ileal oligosaccharide profile, the cecal SCFA profile and protein content, and total tract N retention and metabolizable energy. Digesta from the jejunum (end of pancreatic loop to Meckel's diverticulum), ileum (from Meckel's diverticulum up to 2cm proximal to ileocecal junction), and excreta were also analyzed for starch digestibility to study site-specific starch disappearance based on RSS and RSL.

### Digesta pH Measurements

Digesta samples (one bird per pen) were collected from the duodenum, jejunum, ileum, and caeca for pH determination. The digesta was diluted at the ratio of 1 to 9, with 9ml as distilled water and 1g as digesta, vortexed and stirred with a magnetic stirrer for proper homogenization. The pH was then measured using a sterile glass pH electrode (Thermo Scientific, Beverly, MA).

### Digestive Tract Morphometry and Histomorphology

The digestive tract was excised from one randomly selected bird per cage and sectioned into the crop, proventriculus, gizzard, duodenum (pancreatic loop), jejunum (end of the pancreatic loop to Meckel's diverticulum), ileum (from Meckel's diverticulum to the ileocecal junction) and ceca. The adjoining tissues were carefully removed, and the sections were weighed with the digesta. The weights and lengths of each of the gastrointestinal tract (**GIT)** sections were expressed relative to the body weight.

Two-centimeter sections of mid-jejunum were collected from another 2 randomly selected birds per cage and fixed in 10% neutral buffered formalin for morphological assessment. Subsequently, the samples were dehydrated with ethanol, cleared with sub-x/xylene, embedded in paraffin, and sectioned into 5 μm. The sections were then stained with hematoxylin-eosin stain for morphology measurement. Images of the stained samples were captured at 4 × magnification using BZ microscope (BZ-X800; Keyence Inc., Itasca, IL) and analyzed using BZ-X800 Analyzer. Villus height (the tip of the villus to the villus-crypt junction) and the corresponding crypt depth (villus-crypt junction to the base of the crypt) for 6 villi and crypts per section were determined. The villus height-to-crypt depth ratio was subsequently calculated.

### Chemical Analyses

Diets, excreta, ileal, and jejunal digesta were oven-dried and ground (0.5mm sieve) prior to chemical analyses. Except stated otherwise, all analyses followed the AOAC, 2006; AOAC, 2012) procedure. The dry matter (Method 934.0) was determined by drying the samples at 100°C for a period of 24 h. The N content of the samples was measured with the aid of a combustion nitrogen analyzer (LECO, St. Joseph, MI) using 15 mg of sample. The gross energy was measured using an isoperibol bomb calorimeter (Model 6200, Parr Instruments, Moline, IL), with benzoic acid serving as the calibration standard. Total starch was analyzed using the Megazyme assay kit (**K-AMYL**). These RSS used in the current experiment belong to the RS2 category and are native RS. The RS assay was done by Eurofins Food Testing, Netherlands, using the AOAC method 2011.25. The titanium dioxide contents of the samples were evaluated as outlined by Short et al. (1996).

### Cecal Short-Chain Fatty Acid Profile

A total of 1 g of cecal digesta was diluted with 3-ml deionized water, mixed, and centrifuged (10,000 × *g*) for 10 min. The supernatant (1 mL) was mixed with 0.2 mL 25 % (w/v) metaphosphoric acid solution and frozen overnight. The thawed samples were then centrifuged, and 0.75 mL of the supernatant was mixed with 0.15 mL of internal standard to have 0.9 ml solution. 1.8 ml ethyl acetate was then added and tubes were vortexed for 15 s. The solution was allowed to settle for at least 5 min, and about 1.2 mL of the top layer was transferred into a glass vial, and the SCFA content was measured using gas chromatography (Shimadzu GC-2010 Plus; Shimadzu Corporation, Japan). Results were compared to known standards, and concentrations of SCFA were expressed in m*M* (Lourenco et al., 2020).

### Cecal Protein Analysis

The soluble protein concentration of the cecal content was determined using a BCA protein assay kit, according to the manufacturer's instructions (EMD Millipore Corp, MA).

### Relative Ileal Oligosaccharide Profile Analysis

Matrix-assisted laser desorption ionization mass spectrometry was used for the evaluation of the ileal digesta oligosaccharide profile, as outlined by Lin and Olukosi (2021). Briefly, the ileal digesta from 3 randomly selected birds were collected, stored at -20°C prior to freeze-drying. About 30 mg of the digesta sample was dispersed in 7 mL of 80% ethanol, chilled at 4°C for 1 h, and centrifuged at 4°C for 20 min at 1,200 × *g*. The supernatant was dried by a stream of nitrogen, weighed, and then diluted with 1 mL of water. Approximately 100 μL of the diluted sample was then freeze-dried in a small glass vial overnight. The dried sample was permethylated using dimethyl sulfoxide and dichloromethane, washed with water, dried, dissolved in methanol, and crystallized with ⊍-dihydroxybenzoic acid. The relative abundance of each oligosaccharide was then determined using the relative intensity from matrix-assisted laser desorption ionization mass spectrometry (**MALDI- MS**) detection (Applied Biosystems, MDS Analytical Technologies). The abundance of each sugar was calculated relative to the total intensity of the sugars evaluated in percentage.

### Quantitative Real-Time PCR Analysis

Total RNA extraction was done using QiAzol lysis reagent (QIAGEN, Hilden, Germany (Invitrogen, Carlsbad, CA). The RNAs extracted were cleaned using RNeasy Mini Kit (Qiagen, Valencia, CA) as outlined by the manufacturer. The concentrations were measured with NanoDrop 2000 Spectrophotometer (Thermo Scientific, Wilmington, DE) and adjusted to 200 ng/mL with nuclease-free water. Ten μL of the RNAs were reverse transcribed to cDNA in a 20 μL reaction volume using a high-capacity cDNA reverse transcription kit (Thermo Fischer Scientific, Waltham, MA). The cDNA was diluted to 20 ng/μL following concentration measurement and the RT-qPCR was done using StepOnePlus (Applied Biosystems, Carlsbad, CA) with reaction master mix iTaq Universal SYBR Green Supermix (Bio-Rad, Hercules, CA). The housekeeping gene used was β-actin and the fold change was calculated using the 2^−∆∆CT^ method (Livak and Schmittgen, 2001). The primer sequence and their functions are presented in Table 4.

**Table 4:** List of primers and their functions.

Symbol	Gene name	Function	Forward primer	Reverse primer
βeta-actin	β-actin	Housekeeping gene	ACCGGACTGTTACCAACACC	GACTGCTGCTGACACCTTCA
MUC2	Mucin 2	Mucin secretion	ATGCGATGTTAACACAGGACTC	GTGGAGCACAGCAGACTTTG
OCLN	Occludin	Tight junction	CTGCTCTGCCTCATCTGCTTCTTC	CCATCCGCCACGTTCTTCACC
GLUT- 2	Glucose transporter- 2	Glucose transporter	TCATTGTAGCTGAGCTGTT	CGAAGACAACGAACACATAC
PepT1	Peptide transporter- 1	Peptide transporter	CCCCTGAGGAGGATCACTGTT	CAAAAGACCAGCAGCAACGA
FFAR2	Free-fatty acid receptor – 2	Free-fatty acid receptor	AACGCCAACCTCAACAACTC	TGGGAGAAGTCATCGTAGCA
FFAR3	Free-fatty acid receptor – 3	Free-fatty acid receptor	GAAGGTGGTTTGGGAGTGAA	CAGAGGATTTGAGGCTGGAG
MCT4	Monocarboxylate transporter 4	SCFA transporters	GCTGGTCTCAAGTGGGTTAG	CCACCGTAATCGACAGACATAG
MCT1	Monocarboxylate transporter 1	SCFA transporters	AGCAGCATCCTGGTGAACAAG	AGGCACCCACCCACGAT

### Calculations

All calculations are presented on a dry matter basis. The total tract nutrient retention and apparent ileal digestibility of starch and energy, and DM were calculated using the index method by the following equations:

Digestibility(%)=100×[1−((Ci/Co)×(No/Ni))]

DMDigestibility(%)=100×[1−(Ci/Co)]

Where Ci and Co are the % concentrations of titanium dioxide in the diet and excreta/digesta, respectively; Ni and No are the % nutrient contents in the diet and excreta/digesta, respectively.

Nitrogen-corrected apparent metabolizable energy (**AMEn**) and apparent metabolizable energy (**AME**) in kcal/kg were calculated as follows:

AME=GEi−[(Ci/Co)×(GEo)]

AMEn=AME−[8.22×(NR/DMI)]

where GEi and GEo are gross energy contents (kcal/kg) of the diets and excreta, respectively. The NR is the retained nitrogen (g) and DMI is the dry matter intake (g) and calculated as follows:

NR(g)=(NI−NO)

DMI(g)=Feedintake,g×FeedDM(coefficient)

Where NI and NO are nitrogen intake (g) and output (g), respectively.

### Statistical Analysis

The statistical model for the experiment was:

$$Y_{ijkl}=\mu +\alpha_{i}+\beta_{j}+{\left[\beta \left(\gamma \right)\right]}_{jk}\;{\left[\beta \left(\delta \right)\right]}_{jl}+{\left[\beta \left(\gamma \delta \right)\right]}_{jkl}+\varepsilon_{ijkl}$$

where Y*ijk*l is the response variable, *μ* is the overall mean, *α*_i_ is the random effect of *i*th block, *β*_j_ is the fixed effect of *j*th treatment group, *β*(*γ*)_jk_ represent the fixed effect of *k*th resistant starch source within *j*th treatment group, *β*(*δ*)_jl_ represents the fixed effect of *l*th resistant starch level within *j*th treatment group. *β*(*γ*δ)_jkl_ *is the* interaction effect of *k*th resistant starch source and *l*th resistant starch level within *j*th treatment group, and the *ε_ijk_*_l_ is the residual error. Data were analyzed using the mixed model procedure of JMP Pro 13.2.0 (SAS Institute Inc., Cary, NC) in a 3 × 3 + 1 factorial arrangement. The factors were 3 resistant starch sources, each at 3 inclusion levels nested with the control diet. Means with significant differences were separated using Tukey's HSD, and the significant P-value was set at P ≤ 0.05. Main effects are discussed when there are no significant interactions, whereas the simple effects are described in cases of significant interactions.

## RESULTS

### Growth Performance

In the starter phase, RSS × RSL was significant (*P* < 0.05) for FCR (Table 5). The FCR of birds receiving BS or RPS was not influenced by inclusion levels, whereas birds receiving 50 g/kg HCS had higher FCR than other treatments except RPS at 100 g/kg. There were significant main effects of RSL and RSS (*P* < 0.05) for the WG and FI, respectively. Birds fed 100 g/kg RS had higher (*P* = 0.034) WG than 50 g/kg. The feed intake was greater (*P* = 0.05) in the birds that received BS and RPS diets and similar across all the other diets. During the grower phase, there was a significant (*P* < 0.05) RSS × RSL for FI. Birds fed the 100 g/kg RPS diet had higher FI (*P* = 0.042) than 25 g/kg BS and the control diet. The main effects of RSS and RSL were significant (*P* < 0.05) for FCR. Birds that received BS diets had increased FCR (*P* = 0.018) than other treatments, whereas the birds that received 100 g/kg RS had greater (*P* = 0.045) FCR than others. In the overall phase, the main effects of RSS were significant for FI and FCR (*P* < 0.05). Birds fed the RPS diets had higher (*P* = 0.011) FI than those that received HCS or control diets. On the other hand, FCR was greater (*P* = 0.030) for birds that received BS diets than for all other diets. There were no significant treatment effects on the final body weight.

**Table 5:** Growth performance response of the broiler chickens receiving dietary resistant starches from different sources fed at different levels up to d 21.

RSL, g/kg	RSS	Starter phase (d 0–8)			Grower (d 8–21)			Overall (d 0–21)			
		WG, g	FI, g	FCR	WG, g	FI, g	FCR	WG	FI, g	FCR	FBW, g
Control (0)		114^AB^	159^ab^	1.40^b^	717	975^b^	1.50^bB^	831	1134^b^	1.47^b^	874
Main effects means of resistant starch source (**RSS**)											
	BS	117	170^a^	1.46	633	1030	1.67^a^	750	1199^ab^	1.63^a^	793
	RPS	113	167^a^	1.50	730	1066	1.50^b^	843	1233^a^	1.49^b^	886
	HCS	106	158^ab^	1.52	666	996	1.51^b^	772	1154^b^	1.50^b^	816
Main effects means of resistant starch level (**RSL**)											
25		113^AB^	163	1.46	692	1009	1.51^B^	805	1172	1.49	849
50		105^B^	162	1.57	686	1037	1.52^B^	791	1199	1.52	835
100		118^A^	170	1.46	651	1046	1.66^A^	769	1216	1.61	812
Pooled SEM		4.1	4.2	0.030	35	18	0.050	34	21	0.046	34
Simple effect means											
25	BS	115	165	1.44^b^	645	1005^b^	1.58	760	1170	1.55	804
50	BS	114	170	1.50^b^	681	1072^ab^	1.58	795	1242	1.57	838
100	BS	122	174	1.44^b^	572	1012^ab^	1.86	694	1186	1.77	738
25	RPS	112	164	1.50^b^	782	1028^ab^	1.40	894	1192	1.40	937
50	RPS	112	165	1.49^b^	680	1033^ab^	1.53	792	1199	1.52	837
100	RPS	114	173	1.53^ab^	728	1136^a^	1.57	842	1309	1.56	885
25	HCS	111	160	1.45^b^	650	995^b^	1.54	762	1154	1.52	806
50	HCS	89	151	1.72^a^	697	1005^b^	1.44	786	1156	1.47	830
100	HCS	118	163	1.41^b^	652	989^b^	1.54	770	1152	1.52	814
Pooled SEM		5.9	6.1	0.040	52	28	0.080	51	31	0.068	501
Probabilities											
RSS		0.092	0.050	0.158	0.071	0.012	0.018	0.074	0.011	0.030	0.074
RSL		0.034	0.218	0.002	0.568	0.253	0.045	0.678	0.227	0.069	0.676
RSS × RSL		0.155	0.829	0.001	0.463	0.042	0.342	0.512	0.075	0.403	0.514

RSS, resistant starch source; RSL, resistant starch level; BS, Banana starch; RPS, raw potato starch; HCS, high-amylose corn starch; WG, body weight gain; FI, feed Intake; FCR, feed conversion ratio; FBW, final body weight. n = 8 replicate cages per treatment.

AB,ab : Means in a column, within a group, with different superscripts differ significantly (P ≤ 0.05). Small letter superscripts compare the RSS group nested with control, or the simple effect means nested with control (in cases of significant interaction). Block letter superscripts compare RSL nested with control.

### Nutrient Digestibility, Total Tract Retention, and Metabolizable Energy

There were significant (*P* < 0.05) RSS × RSL for all the responses on d 21 (Table 6). For jejunal DM, digestibility was greater (*P* = 0.01) in the control diet than all levels of BS, 100 g/kg RPS and 25 g/kg HCS. Jejunal starch digestibility was greater (*P* < 0.001) in the control diet than all levels of BS and 50 and 100 g/kg RPS. Jejunal starch digestibility decreased (*P* < 0.05) with increasing RSL for all RS except for HCS.

**Table 6:** Apparent jejunal and ileal nutrient digestibility, total tract nutrient retention, and dietary metabolizable energy on d 21 for the broiler chickens receiving dietary resistant starches from different sources fed at different levels.

RSL,	RSS	Jejunum		Ileum			Total tract			
g/kg		DM	Starch	DM	N	Starch	N	Starch	AME, kcal/kg	AMEn, kcal/kg
Control (0)		62.0^a^	96.0^a^	77.8^a^	81.3^a^	98.57^a^	67.6^a^	96.4^a^	3428^ab^	3352^ab^
Main effects means of resistant starch source (**RSS**)										
	BS	44.1	88.7	66.2	72.0	91.25	53.2	87.6	3044	2935
	RPS	53.5	88.6	72.6	79.2	91.1	63.2	88.5	3249	3160
	HCS	54.0	93.2	72.8	77.73	95.4	61.5	91.7	3293	3199
Main effects means of resistant starch level (**RSL**)										
25		49.6	92.7	71.0	75.2	95.4	58.4	92.1	3180	3082
50		50.0	90.6	70.2	75.4	92.8	59.3	90.1	3193	3096
100		51.6	87.3	70.5	70.5	89.5	60.3	85.6	3212	3117
Pooled SEM		1.52	0.524	0.76	1.33	0.38	1.20	0.33	20.0	22.0
Simple effect means										
25	BS	43.9^d^	91.9^bcd^	68.7^cde^	73.6^ab^	95.3^b^	57.6^bc^	92.5^b^	3138^de^	3040^de^
50	BS	43.8^d^	89.6^cd^	63.8^e^	67.3^b^	91.1^c^	53.8^cd^	88.2^d^	3006^ef^	2897^ef^
100	BS	44.6^d^	84.6^e^	66.1^de^	75.0^ab^	87.4^d^	48.3^d^	82.2^e^	2987^f^	2869^f^
25	RPS	57.2^abc^	92.9^abc^	73.9^ab^	79.2^a^	95.2^b^	61.4^abc^	92.1^bc^	3244^cd^	3152^cd^
50	RPS	52.4^abcd^	88.2^d^	73.3^abc^	78.9^a^	91.7^c^	62.4^ab^	90.2^cd^	3321^bc^	3232^bc^
100	RPS	51.0^bcd^	84.7^e^	70.3^bcd^	79.4^a^	86.3^d^	65.8^a^	83.2^e^	3181^d^	3097^cd^
25	HCS	47.8^cd^	93.2^ab^	70.3^bcd^	72.7^ab^	95.8^b^	56.3^bcd^	91.7^bc^	3158^d^	3054^d^
50	HCS	55.2^abc^	93.9^ab^	73.4^abc^	80.0^a^	95.7^b^	61.6^abc^	92.0^bc^	3252^cd^	3160^cd^
100	HCS	59.2^ab^	92.5^abc^	74.9^ab^	80.5^a^	94.8^b^	66.7^a^	91.3^bc^	3469^a^	3384^a^
Pooled SEM		2.24	0.77	1.11	1.95	0.55	1.76	0.48	29.4	32.3
Probabilities										
RSS		< 0.001	< 0.001	< 0.001	< 0.001	< 0.001	< 0.001	< 0.001	< 0.001	< 0.001
RSL		0.555	< 0.001	0.680	0.097	< 0.001	0.433	< 0.001	0.403	0.424
RSS × RSL		0.005	< 0.001	< 0.001	0.012	< 0.001	< 0.001	< 0.001	< 0.001	< 0.001

RSS, resistant starch source; RSL, resistant starch level; BS, Banana starch; RPS, raw potato starch; HCS, high-amylose corn starch; DM, dry matter; N, nitrogen.

n = 8 replicate cages per treatment.

AB, ab : Means in a column, within a group, but with different superscripts differ significantly (*P* ≤ 0.05). Small letter superscripts compare the RSS group nested with control, or the simple effect means nested with control (in cases of significant interaction). Block letter superscript compares RSL nested with control.

For apparent ileal nutrient digestibility, the DM digestibility was greater (*P* < 0.001) in birds fed the control diet than in all levels of BS, 100 g/kg RPS, and 25 g/kg HCS. Nitrogen digestibility was greater (*P* = 0.01) in the control diet, all levels of RPS, and 50 and 100 g/kg HCS than in 50 g/kg BS diets. The ileal starch digestibility was greater (*P* < 0.001) in the control diet than in all RS diets. The ileal starch digestibility decreased as the RSL increased for all RS except for HCS.

For total tract nutrient utilization, N retention was greater (*P* < 0.001) in birds fed the control diet, 100 g/kg RPS, and 100 g/kg HCS diets than all levels of BS and 25 g/kg HCS. The N retention increased with increasing RPS and HCS levels but decreased with increasing levels of BS. Starch retention was greater (*P* < 0.001) in the control diet than in all RS diets. The AME and AMEn were greater (*P* < 0.001) in birds fed 100 g/kg HCS diet than in other RS diets, and both decreased with increasing RSL for all RS except HCS.

### Relative Ileal Oligosaccharides Profile

There were significant RSS × RSL ( < 0.05) for the relative abundance of Hex (3) and Pent (3) (Table 7). The relative abundance of Hex (3) was greater (*P* = 0.01) in birds fed 50 g/kg BS, 100 g/kg RPS, 25 and 100 g/kg HCS and the control diets. Birds fed 50g/kg HCS had greater (*P* = 0.001) relative abundance of Pent (3) than 50 and 100 g/kg BS, 25 and 100 g/kg RPS and HCS. Pent (3) had greater relative abundance (*P* = 0.001) in birds fed 50 g/kg of RPS and HCS than other inclusion levels except BS which had higher relative abundance of Pent (3) at 25 g/kg. There was a significant RSL main effect (P = 0.01) on Hex (5) relative abundance, with birds fed 50 g/kg RS diets having lower relative abundance than the control and 100 g/kg RS.

**Table 7:** Relative ileal oligosaccharide profile (%) on d 21 for the broiler chickens receiving dietary resistant starches from different sources fed at different levels.

RSL (g/kg)	RSS	Hex(3)	Hex(4)	Hex(5)	Hex(6)	Pent(3)	Pent(4)	Pent(5)	Pent(6)
Control		35.9a	42.9	13.6^A^	0.99	1.53^b^	0.00	2.73	0.00
Main effects means of resistant starch source (RSS)									
	BS	30.4	30.8	6.28	0.59	3.63	0.02	4.19	0.00
	RPS	29.5	28.4	7.83	1.28	2.56	0.10	4.10	0.33
	HCS	32.7	36.1	7.97	0.60	4.38	0.00	5.26	0.15
Main effects means of resistant starch level (RSL)									
25		29.6	32.3	8.24^AB^	1.04	2.89	0.12	3.76	0.07
50		26.9	28.0	4.60^B^	0.67	5.35	0.00	4.93	0.35
100		36.1	35.1	9.25^A^	0.76	2.34	0.00	4.85	0.06
Pooled SEM		3.45	3.98	1.751	0.550	0.897	0.081	1.588	0.170
Simple effect means									
25	BS	20.3^c^	26.1	5.23	0.02	5.42^ab^	0.05	3.88	0.00
50	BS	36.9^a^	38.4	4.08	0.58	1.81^b^	0.00	3.17	0.00
100	BS	33.9^ab^	28.0	9.54	1.18	3.67^b^	0.00	5.51	0.00
25	RPS	30.1^b^	29.6	8.53	2.45	1.54^b^	0.31	3.50	0.21
50	RPS	20.1^c^	22.2	4.63	0.74	4.88^ab^	0.00	4.71	0.61
100	RPS	38.2^a^	33.5	10.3	0.63	1.26^b^	0.00	4.09	0.18
25	HCS	38.3^a^	41.2	11.0	0.65	1.71^b^	0.00	3.92	0.00
50	HCS	23.7^c^	23.4	5.07	0.68	9.37^a^	0.00	6.92	0.45
100	HCS	36.1^a^	43.7	7.87	0.47	2.08^b^	0.00	4.96	0.00
Pooled SEM		4.93	5.69	2.501	0.786	1.282	0.115	2.269	0.243
Probabilities									
RSS		0.724	0.317	0.809	0.777	0.468	0.378	0.926	0.302
RSL		0.099	0.079	0.011	0.359	0.001	0.411	0.645	0.123
RSS × RSL		0.010	0.112	0.212	0.150	0.001	0.543	0.454	0.618

Hex, hexoses with the indicated number of monomers; Pent, pentoses with the indicated number of monomers.

n = 8 replicate cages per treatment.

AB,ab : Means in a column, within a group, but with different superscripts differ significantly (P ≤ 0.05). Small letter superscripts compare the RSS group nested with control, or the simple effect means nested with control (in cases of significant interaction). Block letter superscript compares RSL nested with control.

### Cecal Short-Chain Fatty Acids Profile

The main effects of RSL were significant (*P* < 0.05) for all the cecal SCFA analyzed and there was a significant main effect of RSS and RSL for total SCFA (Table 8). The individual and total SCFA concentrations (*P* < 0.05) were increased by 25 g/kg and 100 g/kg RS diets. Isobutyrate and isovalerate concentrations were greater (*P* < 0.05) at 25 g/kg than at 50 g/kg RSL. The birds that received HCS diets had elevated (*P* = 0.029) cecal total SCFA than those fed RPS diets.

**Table 8:** Cecal short-chain fatty acids profile (**mM**) on d 21 for the broiler chickens receiving dietary resistant starches from different sources fed at different levels.

RSL, g/kg	RSS	Acetate	Propionate	Isobutyrate	Butyrate	Isovalerate	Valerate	Total SCFA
Control (0)	87.0^AB^	5.45^AB^	0.48^AB^	20.9^AB^	0.55^AB^	1.20^AB^	116^ab^^AB^	
Main effects means of resistant starch source (RSS)								
	BS	89.4	5.62	0.48	20.0	0.58	1.18	117^ab^
	RPS	83.4	5.17	0.48	19.9	0.53	1.11	111^b^
	HCS	94.2	5.87	0.51	22.2	0.54	1.30	125^a^
Main effects means of resistant starch level (RSL)								
25		88.4^AB^	6.13^A^	0.56^A^	21.7^A^	0.62^A^	1.27^A^	119^AB^
50		81.5^B^	4.57^B^	0.38^B^	18.5^B^	0.45^B^	1.04^B^	107^B^
100		97.2^A^	5.97^A^	0.53^AB^	21.9^A^	0.58^AB^	1.28^A^	127^A^
Pooled SEM		4.02	0.404	0.049	1.13	0.053	0.082	4.4
Simple effect means								
25	BS	89.8	6.79	0.51	21.6	0.58	1.25	121
50	BS	78.7	4.7	0.48	16.5	0.56	1.06	102
100	BS	99.8	5.38	0.47	21.9	0.60	1.23	129
25	RPS	85.2	5.64	0.55	20.1	0.58	1.20	113
50	RPS	78.1	3.87	0.32	19.5	0.41	0.95	103
100	RPS	86.9	5.99	0.58	20.0	0.61	1.18	115
25	HCS	90.0	5.95	0.64	23.3	0.70	1.37	122
50	HCS	87.8	5.15	0.35	19.4	0.40	1.10	114
100	HCS	105.0	6.52	0.54	23.8	0.53	1.45	138
PooledSEM		5.89	0.59	0.072	1.65	0.078	0.120	6.3
Probabilities								
RSS		0.087	0.340	0.857	0.165	0.782	0.143	0.029
RSL		0.007	0.003	0.008	0.024	0.032	0.022	0.001
RSS × RSL		0.742	0.400	0.285	0.558	0.386	0.960	0.667

RSS, resistant starch source; RSL, resistant starch level; BS, Banana starch; RPS, raw potato starch; HCS, high-amylose corn starch.

n = 8 replicate cages per treatment.

AB,ab : Means in a column, within a group, but with different superscripts differ significantly (P ≤ 0.05). Small letter superscripts compare the RSS group nested with control, or the simple effect means nested with control (in cases of significant interaction). Block letter superscript compares RSL nested with control.

### Intestinal Digesta pH and Cecal Protein

There were no significant treatment effects on the duodenal, jejunal, ileal, and cecal digesta pH in this experiment (Table 9). However, the main effects of RSS and RSL were significant (*P* < 0.05) for cecal protein. Dietary BS and HCS reduced (*P* = 0.001) the cecal protein and in addition, 25 g/kg RS diets reduced (*P* = 0.03) the cecal protein more than the other RS levels.

**Table 9:** Digesta pH along the digestive tract and cecal soluble protein on d 21 for the broiler chickens receiving dietary resistant starches from different sources fed at different levels.

RSL (g/kg)	RSS	Duodenum pH	Jejunum pH	Ileum pH	Cecal pH	Cecal protein (µg/g DM)
Control (0)		6.31	6.34	6.67	6.70	214^aA^
Main effects means of resistant starch source (**RSS**)						
	BS	6.31	6.32	6.62	6.55	169^b^
	RPS	6.35	6.34	6.67	6.51	193^a^
	HCS	6.34	6.30	6.78	6.60	158^b^
Main effects means of resistant starch level (**RSL**)						
25		6.35	6.36	6.59	6.54	158^B^
50		6.34	6.33	6.81	6.47	181^AB^
100		6.29	6.28	6.73	6.54	180^AB^
Pooled SEM		0.027	0.023	0.142	0.123	7.4
Simple effect means						
25	BS	6.33	6.36	6.41	6.44	138
50	BS	6.35	6.32	6.79	6.69	193
100	BS	6.26	6.29	6.66	6.53	176
25	RPS	6.38	6.36	6.61	6.76	189
50	RPS	6.35	6.33	6.70	6.27	197
100	RPS	6.32	6.34	6.69	6.49	193
25	HCS	6.39	6.35	6.55	6.77	148
50	HCS	6.33	6.33	6.95	6.45	153
100	HCS	6.29	6.22	6.85	6.60	172
Pooled SEM		0.040	0.034	0.208	0.180	10.6
Probabilities						
RSS		0.440	0.822	0.942	0.341	0.001
RSL		0.127	0.097	0.203	0.211	0.027
RSS × RSL		0.823	0.274	0.521	0.155	0.073

RSS, resistant starch source; RSL, resistant starch level; BS, Banana starch; RPS, raw potato starch; HCS, high-amylose corn starch.

n = 8 replicate cages per treatment.

AB,ab : Means in a column, within a group, with different superscripts differ significantly (P ≤ 0.05). Small letter superscripts compare the RSS group nested with control, or the simple effect means nested with control (in cases of significant interaction). Block letter superscript compares RSL nested with control.

### Digestive Organs Index

The main effect of RSL was significant (*P* < 0.05) for relative duodenal weight and cecal weights whereas the RSS × RSL was only significant for relative ileal weight (Table 10). The relative weight of duodenum generally increased (*P* < 0.037) with increasing RSL. Birds fed the 100 g/kg BS diet had higher (*P* = 0.03) relative ileal length than those fed 25 g/kg RPS, and this increased with increasing BS level. The relative cecal weight was greater (*P* = 0.01) in birds fed 100 g/kg RS than 25 and 50 g/kg RS.

**Table 10:** Digestive organ index on d 21 for the broiler chickens receiving dietary resistant starches from different sources fed at different levels.

RSL, g/kg	RSS	Crop	Proventriculus	Gizzard	Duodenum		Jejunum		Ileum		Ceca
		W	W	W	W	L	W	L	W	L	W
Control (0)		3.14	5.07	20.8	28.6^AB^	9.71	67.1	18.8	67.5^ab^	14.6	4.21^AB^
Main effects of resistant starch source (**RSS**)											
	BS	3.06	5.49	23.6	29.8	10.6	65.2	18.9	72	15.0	4.31
	RPS	3.02	5.33	24.1	28.6	10.5	65.8	19.0	68.1	15.7	4.33
	HCS	3.26	5.56	23.7	29.3	11.0	66.0	18.5	71.7	14.8	4.20
Main effects of resistant starch level (**RSL**)											
25		3.01	5.50	22.7	27.9^AB^	9.87	64.0	18.1	67.8	14.6	4.03^B^
50		3.11	5.28	24.3	29.1^AB^	11.2	67.7	20.2	70.5	15.8	4.14^B^
100		3.22	5.6	24.5	30.7^A^	11	65.3	18.2	73.5	15.1	4.70^A^
Pooled SEM		0.135	0.248	0.76	1.021	0.405	2.77	0.76	3.24	0.67	0.19
Simple effect means											
	BS	2.95	5.34	22.6	27.5	10.1	63.5	18.7	64.8^ab^	14.7	3.74
	BS	3.29	5.44	23.2	27.9	10.2	65.6	19.1	68.9^ab^	15.4	3.95
	BS	2.93	5.68	25.1	34.0	11.5	66.6	18.9	82.4^a^	15.1	5.23
	RPS	2.89	5.63	21.9	27.1	9.41	61.0	18.1	60.0^b^	14.1	4.10
	RPS	3.13	5.34	26	31.2	11.7	76.0	21.4	79.5^ab^	16.9	4.39
	RPS	3.03	5.68	24.5	27.4	10.4	60.4	17.5	64.9^ab^	16.1	4.48
	HCS	3.18	5.54	23.5	29.1	10.1	67.7	17.4	78.7^ab^	15.0	4.24
	HCS	2.91	5.70	23.6	28.1	11.8	61.6	20.0	63.0^ab^	15.1	4.09
	HCS	3.70	5.45	23.9	30.7	11.0	68.9	18.1	73.4^ab^	14.2	4.28
Pooled SEM		0.198	0.364	1.111	1.498	0.590	4.058	1.125	4.760	0.976	0.277
Probabilities											
RSS		0.077	0.735	0.642	0.149	0.133	0.599	0.586	0.125	0.936	0.877
RSL		0.277	0.504	0.110	0.037	0.068	0.93	0.429	0.202	0.776	0.008
RSS × RSL		0.089	0.928	0.850	0.167	0.431	0.487	0.804	0.027	0.635	0.095

RSS, resistant starch source; RSL, resistant starch level; BS, Banana starch; RPS, raw potato starch; HCS, high-amylose corn starch.

W, weight (g/kg body weight); L, length cm/kg body weight).

n = 8 replicate cages per treatment.

AB,ab : Means in a column, within a group, with different superscripts differ significantly (*P* ≤ 0.05). Small letter superscripts compare the RSS group nested with control, or the simple effect means nested with control (in cases of significant interaction). Block letter superscript compares RSL nested with control.

### Jejunal Histomorphology

There were no significant RSS × RSL or main effects for jejunal histomorphology except for the significant RSS effect on crypt depth (Table 11). Birds that received RPS and HCS diets had deeper (P < 0.01) jejunal crypts than those that received the BS diets.

**Table 11:** Jejunal histomorphology on d 21 for the broiler chickens receiving dietary resistant starches from different sources fed at different levels.

RSL, g/kg	RSS	VH, µm	CD, µm	VW, µm	VH/CD
Control (0)		1,361	206^ab^	220	6.72
Main effects means of resistant starch source (**RSS**)					
	BS	1,510	219^b^	229	6.94
	RPS	1,581	255^a^	243	6.40
	HCS	1,702	256^a^	244	6.82
Main effects means of resistant starch level (**RSL**)					
25		1,514	241	245	6.43
50		1,600	242	249	6.75
100		1,679	248	223	6.98
Pooled SEM		72	11.5	11.3	0.355
Simple effect means					
25	BS	1,413	211	232	6.71
50	BS	1,579	233	232	6.83
100	BS	1,537	212	224	7.29
25	RPS	1,461	245	252	6.14
50	RPS	1,491	236	241	6.49
100	RPS	1,793	285	236	6.57
25	HCS	1,669	267	251	6.44
50	HCS	1,729	255	275	6.93
100	HCS	1,707	245	208	7.08
Pooled SEM		104	16.8	16.5	0.517
Probabilities					
RSS		0.076	0.013	0.474	0.358
RSL		0.157	0.861	0.116	0.434
RSS × RSL		0.325	0.173	0.356	0.996

RSS, resistant starch source; RSL, resistant starch level; BS, Banana starch; RPS, raw potato starch; HCS, high-amylose corn starch; VH, villi height; CD, crypt depth; VW, villi width.

n = 8 replicate cages per treatment.

AB,ab : Means in a column, within a group, with different superscripts differ significantly (P ≤ 0.05). Small letter superscripts compare the RSS group nested with control, or the simple effect means nested with control (in cases of significant interaction). Block letter superscript compares RSL nested with control.

### Jejunal mRNA Expression of Glucose and Peptide Transporters and Epithelial Integrity Genes and Cecal Expression of Fatty Acid Transporters

The jejunal GLUT-2 mRNA expression was greater (*P* = 0.02) in birds that received RPS or BS than those fed HCS diet (Table 12). There was significant RSS × RSL for jejunal PEPT1 relative expression. Generally, relative expression of PEPT1 increased (*P* < 0.05) with increasing BS level but decreased with increasing level of RPS or HCS. The relative mRNA expression of jejunal MUC-2 was lower (*P* < 0.05) for birds that received BS and HCS than the control diet. The main effects of RSL were significant (*P* < 0.05) for cecal FFAR2 and MCT4. Birds fed 25 g/kg RS had greater (*P* = 0.003) mRNA expression of FFAR2 than those fed the 100 g/kg RS diet. On the other hand, birds fed 50 g/kg RS or the control diet had greater (*P* = 0.05) mRNA expression of MCT4 than those fed 100 g/kg. The RSS × RSL was significant (*P* < 0.05) for cecal MCT1. Birds fed the control diet had higher (*P* = 0.001) mRNA expression of MCT1. Among RS diets, birds fed 25 and 100 g/kg BS had greater (P < 0.05) MCT1 expression than 50 g/kg BS, and those fed 50 g/kg HCS had greater (P < 0.05) relative expression MCT1 than those fed 100 g/kg.

**Table 12:** Relative mRNA expression of jejunal nutrient transporters and epithelial integrity and cecal fatty acid transporters on d 21 for the broiler chickens receiving dietary resistant starches from different sources fed at different levels.

		Jejunum	Caeca						
RSL, g/kg	RSS	GLUT 2	PEPT1	MUC 2	Occludin	FFAR2	FFAR3	MCT4	MCT1
Control (0)		1.00^ab^	1.00^b^	1.00^a^	1.00	1.00^AB^	1.00	1.00^A^	1.00^a^
Main effects means of resistant starch source (**RSS**)									
	BS	1.24^a^	1.18	0.67^b^	1.16	1.36	0.78	0.85	0.52
	RPS	1.12^a^	1.13	0.89^ab^	1.09	1.05	0.72	0.89	0.36
	HCS	0.65^b^	1.01	0.68^b^	0.98	1.47	0.9	1.21	0.51
Main effects means of resistant starch level (**RSL**)									
25		0.86	1.08	0.77	1.04	1.71^A^	0.81	0.94^AB^	0.51
50		1.07	1.16	0.81	1.19	1.27^AB^	0.91	1.18^A^	0.46
100		1.09	1.08	0.66	0.99	0.91^B^	0.67	0.82^B^	0.42
Pooled SEM		0.149	0.129	0.081	0.099	0.214	0.126	0.160	0.070
Simple effect means									
25	BS	0.84	0.80^bc^	0.52	1.24	1.28	1.11	0.86	0.63^abc^
50	BS	1.46	1.32^a^	0.83	1.45	1.77	0.66	0.77	0.25^c^
100	BS	1.43	1.42^a^	0.65	0.8	1.04	0.57	0.91	0.68^ab^
25	RPS	1.08	1.32^a^	1.14	0.98	1.45	0.42	0.78	0.41^bc^
50	RPS	1.07	1.20^ab^	0.89	1.2	0.69	0.99	0.97	0.36^bc^
100	RPS	1.22	0.88^b^	0.64	1.09	1.01	0.75	0.93	0.32^c^
25	HCS	0.66	1.14^ab^	0.66	0.91	2.41	0.91	1.18	0.50^bc^
50	HCS	0.68	0.96^b^	0.71	0.93	1.34	1.08	1.81	0.76^ab^
100	HCS	0.62	0.94^b^	0.67	1.08	0.67	0.71	0.63	0.26^c^
Pooled SEM		0.218	0.189	0.118	0.145	0.332	0.195	0.248	0.109
Probabilities									
RSS		0.023	0.485	0.031	0.503	0.189	0.492	0.204	0.119
RSL		0.766	0.730	0.206	0.275	0.003	0.421	0.050	0.349
RSS ×´ RSL		0.319	0.042	0.103	0.128	0.079	0.083	0.079	0.001

RSS, resistant starch source; RSL, resistant starch level; BS, Banana starch; RPS, raw potato starch; HCS, high-amylose corn starch.

n = 8 replicate cages per treatment.

AB,ab : Means in a column, within a group, with different superscripts differ significantly (P ≤ 0.05). Small letter superscripts compare the RSS group nested with control, or the simple effect means nested with control (in cases of significant interaction). Block letter superscript compares RSL nested with control.

## DISCUSSION

Fermentation of RS can enhance gut health through the production of microbial metabolites such as short-chain fatty acids (**SCFAs)**. However, the growth performance of the host organism may be compromised depending on various factors such as the source, concentrations, and duration of RS feeding, as energy from SCFAs is less efficiently utilized than that from glucose. Some studies demonstrated that the weight gain, feed intake and feed efficiency may decrease (Li et al., 2007; Regmi et al., 2011) or not influenced (Nofrarías et al., 2007; Doti et al., 2014) by RS consumption. Hence, the current study was aimed at investigating the influence of different sources and levels of RS on the growth performance and nutrient utilization in broiler chickens in the starter and grower phases. We, therefore, hypothesized that the source and level of RS influence nutrient utilization with consequences on cecal SCFA production, ileal oligosaccharide profile, intestinal health and ultimately the growth performance of broiler chickens.

### Growth Performance

The observed increase in WG in the starter phase of the current experiment at highest concentration of RS (100 g/kg) was accompanied by higher FI observed at this level and could probably be due to lower caloric density of RS which might have fueled the increased feed intake associated with high level of RS inclusion to compensate for reduced energy intake (Zhou et al., 2008; Higgins, 2014). Furthermore, the results obtained in the grower phase with different sources and levels of RS having varying FI and FCR (reduced FCR with RPS and HCS diets, and increased FI as RPS level increased) could be because of the differences in crystallinity and degree of resistance to pancreatic amylase which ultimately influenced their digestion and subsequent fermentability in the distal GIT. Although, despite the crystallinity pattern shared by HCS and RPS, the former is believed to be more resistant due to its higher amylose content and the additional resistance conferred by its potential to form RS3. On the other hand, BS has a different crystallinity pattern and so is considered to be less resistant (Jane et al., 1999; Giuberti et al., 2015). Some banana cultivars exhibit crystallinity similar to RPS and HCS thus showing cultivar-dependence in crystallinity patterns (Wang et al., 2017).

The results obtained on feed intake correspond with the reports of Martinez-Puig et al. (2003) and Fang et al. (2014) that the inclusion of 25% RPS in growing pigs diet elevated the FI when compared with corn starch. It is also in line with increased BW and FI in 1 to 14-days-old ducks fed RPS supplementation in normal non-phytate phosphorous diets (Xu et al., 2021). This was attributed to the varying ability of RS to stimulate intestinal movement, increase absorption and utilization of nutrients in the intestines. Also, availability of microbial-produced SCFA as energy source could reduce glucose oxidation, mediating the effects of RS on growth performance as the birds in this study had ad libitum access to feed (Van Erp et al., 2020). The increased concentration of total SCFA (including butyrate, propionate and acetate) in this study at 25 and 100 g/kg RS diets further corroborate the increased WG in birds fed 100 g/kg RS diets during the starter phase and 25 g/kg RS that seems optimum for growth performance in the grower phase.

### Nutrient Digestibility, Total Tract Retention and Metabolizable Energy

Our observations on apparent jejunal, ileal nutrient digestibility, AMEn and total tract nutrient retention partially agree with the previous finding whereby increased concentrations of RS lowered the total tract nutrient utilization as well as apparent ileal digestibility of nutrients (Gerrits et al., 2012; Cervantes‐Pahm et al., 2014; Giuberti et al., 2015). The inclusion of RS in broiler diets reduced starch digestibility as expected, although 25 g/kg RPS and all levels of HCS had comparable jejunal starch digestibility to the control diet. Morel et al. (2005) postulated that a 1% dietary increase in RS may reduce the total ileal starch by 0.64 %. The observed decrease in jejunal and ileal starch digestibility as the RSL increased for all RS except for HCS may be associated with different physiochemical properties peculiar to RS of different botanical origins resulting in changes in the rate of nutrient absorption and varying endogenous secretions (Giuberti et al., 2015) but also due to the observation that virtually all the starch in HCS was resistant. Further calculations (supplementary figure) indicating a smaller marginal increase (2.23 %) in starch digestibility between jejunum and ileum for the birds that received the HCS diets than other RS sources suggested that most of the starch digestibility in the birds fed the HCS diet occurred in the jejunum. This may not necessarily indicate a degree of resistance of the RS in HCS but a consequence of HCS having nearly 100% resistant starch by analysis compared to BS and RPS.

Greater N retention that increased with increasing RPS and HCS levels was supported by the lower FCR observed in the birds fed these RSS. In addition, the increase in AME and AMEn with increasing levels of HCS could be due to greater total cecal SCFA production and higher jejunal starch digestibility of HCS. This was corroborated by the general view that different RS or even RS of the same type at the same or varying levels of inclusion may elicit variable metabolic responses with evident implications on fermentation kinetics, growth performance response, energy utilization, and nutrient digestibility due to different crystalline polymorphism and physiochemical properties of the starches (Deng et al., 2010; Bach Knudsen, 2011; Haenen et al., 2013; Giuberti et al., 2015).

### Relative Ileal Digesta Oligosaccharide and Ceca Short-Chain Fatty Acid Profiles

Starch digestibility, as influenced by the level and source of RS fed, could determine the type of oligosaccharides that reached the distal GIT (Wiseman, 2006). The profile of distal oligosaccharide, in turn, dictates the different degree of penetration of bacteria into these starch granules, which is related to granule dimension and surface area. This is crucial for certain bacterial strains because substrates need to be broken down by bacterial hydrolytic enzymes before fermentation. The rate of this depolymerization affects how readily RS and other non-digestible carbohydrates are available for bacterial fermentation (Macfarlane and Macfarlane, 2003; Martinez-Puig et al., 2003). These might be the likely reason why different RSS and RSL, influenced the relative abundance of ileal digesta oligosaccharide profile in this study.

Lin and Olukosi (2021) previously reported that dietary fiber modulated the digesta oligosaccharide concentrations with higher Pent(3), Pent(4), Pent(5) and greater SCFA in protease-only supplemented high-fiber diets than in protease-only supplemented low-fiber diets. In the current study, the greater abundance of Hex(3) (moderate mass and chain length) in the HCS diet than in other RS diets, along with a greater abundance of Pent (3) (smaller mass and short chain length) in the birds fed HCS was accompanied with higher total SCFA in birds receiving this diet. This could be a function of its higher starch digestibility than other RS sources which could have significant impact on the structure of the oligosaccharide that entered the ceca. The structure of these oligosaccharides, in turn, might have increased accessibility to fermentative bacteria evidenced by greater production of SCFA in birds fed HCS diets (Tan et al., 2021). These indicated that not only the amount of RS entering the large intestine that matters (Haenen et al., 2013) but its composition and physical structure are crucial and may affect total SCFA production and individual SCFA profile (Martin et al., 1998; Jonathan et al., 2012).

The SCFA contributes to the normal functioning of the gut by acting on the intestinal musculature and vasculatures through their impacts on metabolism of enterocytes and colonocytes (Elia and Cummings, 2007; Hamer et al., 2008; Regassa and Nyachoti, 2018). The SCFA profile in response to RS feeding in the current experiment is similar to previous studies that reported an increase in SCFA production in pigs and birds fed RS diets (Haenen et al., 2013; Qin et al., 2019; Trachsel et al., 2019). Higher production of SCFA in birds fed HCS diets could be attributed to its characteristic ability to enhance substrate flow to the hindgut thereby facilitating increased fermentative production of SCFA (Bird et al., 2007; Regmi et al., 2011; Fouhse et al., 2015; Tan et al., 2021). Our finding on HCS also aligns with that of Den Besten et al. (2013) who reported an increase in cecal and fecal concentrations of butyrate and total SCFA concentration in pigs fed 63% dietary high amylose starch.

### Digesta pH

Despite the increase in the cecal SCFA production observed in this study, the duodenal, jejunal, ileal, and cecal pH were not significantly affected. Although, Fouhse et al. (2015) and Tan et al. (2021) reported reduced pH through production of SCFA in the hindgut of pigs fed dietary HCS diets and a reduction in the pH of both ileal and cecal digesta were found in pigs fed daily with 0.5 or 1.0% dietary raw potato starch over a 28-d period (Heo et al., 2014), whereas the ileal digesta pH was not significantly different in ducks fed varied levels of RPS diets for a period of 14 and 35 d (Qin et al., 2019). The disparity between these results could be due to differences in experimental conditions, RS sources, concentrations, experimental animal and feeding length used in different studies.

### Cecal Protein

Functional fibers are capable of increasing carbohydrate fermentation relative to protein in the hindgut thereby minimizing the disproportionate production of branched-chain volatile fatty acids, ammonia and hydrogen sulfide which may have toxic potential on colonic cells (Magee et al., 2000; Mu et al., 2016). The decrease in cecal protein observed in RS diets is a pointer to increase in carbohydrate fermentation relative to protein in the ceca and corresponds with reduced cecal protein and N- levels reported in broiler chickens fed prebiotics and xylo-oligosaccharide (Lin et al., 2023). This further corroborates the prebiotic effects of RS in distal GIT. Another possible reason for decreased cecal protein is that, the lower total tract and apparent ileal starch digestibility in birds receiving dietary RS may have increased carbon availability in the ceca, raising the carbon-nitrogen ratio of the substrates that were available for microbial fermentation (Sun et al., 2016).

### Histomorphology of the Jejunum and Digestive Organ Index

The knowledge of digestive organ histomorphology helps explain the impact of RS on nutrient utilization as longer villi is indicative of increased surface area and absorptive capacity and deeper crypt may indicate greater cellular turnover and higher organ index may be relevant for energy expenditure of the digesta tract (Montagne et al., 2003). The deeper jejunal crypt depth observed with RPS and HCS diets may be due to digesta viscosity effect of these RS diet (Guo et al., 2022), the viscosity effect being part of the mechanism behind the reduction of cholesterol level by RS through retardation of the interactions between sugars and fatty acids (Mudgil, 2017). Additionally, the tendency for longer jejunal villi in birds that received HCS diets than other diets indicated increased proliferation of jejunal epithelial cells evidenced by significant greater production of cecal total SCFA and acetate as well as numerically higher propionate and butyrate in birds fed HCS diets than other RS diets and the control.

Generally, high inclusion of RS increased the relative weight of both proximal (duodenum) and distal GIT (caecum) which correspond with the results obtained for SCFA. The increased relative organ weights and lengths by dietary RS in the current study indicated fermentative production of SCFA which promoted the proliferation of mucosal epithelium and the growth of enterocytes, increasing the surface area for nutrient absorption. The results on organ index is in line with previous studies that reported an increase in ileal length in ducks fed 60 to 180 g/kg RPS diets (Qin et al., 2019) and increased in the length of the small intestine in rats fed dietary inclusion of 30% high-amylose corn (Toden et al., 2007). It also agrees with the enlargement of ceca reported in rat fed dietary RS (Faulks, 2003).

### Gene expression in the jejunum and caeca

Resistant starch influences the metabolism of the host by modifying the metabolites present in systemic circulation and the expression of intestinal genes that play crucial roles in gut health and nutrient metabolism (Regassa and Nyachoti, 2018). The transport of glucose (the final product of starch digestion) into the bloodstream is facilitated by GLUT-2 (Wright and Loo, 2000). The upward relative mRNA expression of GLUT-2 in the jejunum of broiler chickens fed diets containing RPS and BS implies the adjustment of their capacity for increased intestinal glucose uptake (Sajilata et al., 2006; Gilbert et al., 2008). Higher expression of jejunal PEPT1 in the birds fed diets containing 50, 100 g/kg BS and 25, 50 g/kg RPS and 25 g/kg HCS suggests that dietary RS may increase luminal peptide uptake as supported by decreased cecal protein in birds fed dietary RS in the current study.

Dietary RS may increase mucin secretion and improve the gut barrier functions (Qin et al., 2019). Higher mRNA expression of MUC-2 in birds fed RPS and control diets partially aligns with the reported increase in the expression of colonic mucin genes in growing and finishing pigs fed dietary 23 and 28% RPS in comparison with a corn starch diet (Zhou et al., 2017). The jejunal mRNA expression of occludin was not significantly affected by RS inclusion in our study, although it was numerically higher in birds fed certain sources and level of RS (BS, RPS and 50 g/kg RS) than in the control diets. However, the positive effect of RS on intestinal mucin secretion and tight junction genes have only been reported in the distal GIT (ileum and ceca) (Zhou et al., 2017; Qin et al., 2023), being the major site of RS fermentation.

The activation of free fatty acid receptors 2 and 3 (FFAR2 and FFAR3, respectively) by SCFA in the gastrointestinal tract stimulate the release of satiety-stimulating hormones that regulate feed intake (Hirasawa et al., 2005). The expression of FFAR3 was comparable between the dietary treatments used in this study. The observed depression in cecal mRNA expression of FFAR2 in birds fed 100 g/kg RS, irrespective of source, corresponded with the results obtained for the feed intake. There was a numerical increase in feed intake with increasing RSL. This suggests that the reduction in the expression of FFAR2 could have inhibited the release of satiety of hormones, thereby preventing the reduction in feed intake at that concentration (Hirasawa et al., 2005; Kaji et al., 2011). Haenen et al. (2013) also observed a comparable expression of FFAR2 and FFAR3 in the pigs fed RS or digestible starch diets regardless of higher cecal and colonic SCFA production in RS-fed pigs. The depression in expression of FFAR2, irrespective of higher production of cecal SCFA in the current study could probably be the consequence of the similar results obtained for both SCFA production and FFAR2 in birds fed high RS diet and the control diets, suggesting that the SCFA produced might not be sufficient to elicit the activation of the receptors and subsequently the satiety hormones.

The uptake of the SCFA from the lumen is facilitated by monocarboxylate transporter 1 (**MCT1**) and across the basolateral membrane by monocarboxylate transporter 4 (**MCT4**) (Den Besten et al., 2013). The results showed that the birds fed moderate RS level (50 g/kg RS) and the control diet had greater and comparable mRNA expression of MCT4 whereas, the MCT1 was more expressed in control diets than all RS diets, although 50 g/kg HCS, 25 and 100 g/kg BS diet favored higher expression of MCT1 than other RS diets. A previous study reported higher expression of MCT1 in the caecum of pigs fed RS-diet relative to digestible starch diets (Haenen et al., 2013). The disparity in these results could be the effects of different animal model, length of feeding, concentrations and sources of RS.

## CONCLUSIONS

The Type-2 RS used in this study differentially modulated the nutrient digestion and energy utilization in broiler chickens based on their botanical origins and dietary concentrations with consequences on the composition and structure of oligosaccharides entering the distal gastrointestinal tract. These have possible consequences on the extent of cecal fermentation and, subsequently, the total SCFA production as well as on individual SCFA profiles, each eliciting variable effects on GIT morphometrics and growth performance responses. Future studies should investigate the impact of prolonged feeding of RS on broiler chickens. In addition, phase-specific effects of individual RS at various concentrations should be evaluated as these would provide a clearer understanding of the influence of individual RS on growth performance and gastrointestinal health metrics.

We acknowledge the limitation of not having the same level of analyzed RS in all the diets using the different RSS in the current experiment. An attempt to achieve the same analyzed RS level in all diets will necessitate including some RSS at impractically high levels. Nevertheless, the effect of increasing the levels of each RSS can be interpreted within its context in as much as increasing the inclusion levels of each RSS was accompanied by a corresponding increase of its analyzed level in the respective diets.

## DISCLOSURES

The authors declare no conflicts of interest.

## Appendix Supplementary materials

Image, application 1
