# Supplementary material for: The interactivity of sources and dietary levels of resistant starches – impact on growth performance, starch, and nutrient digestibility, digesta oligosaccharides profile, cecal microbial metabolites, and indicators of gut health in broiler chickens
Source: Poult Sci. 2024 Sep 24;103(12):104337. doi: 10.1016/j.psj.2024.104337 (PMC11752116; doi:10.1016/j.psj.2024.104337)
Supplement: Supplementary file 1 [file mmc1.pdf]

Supplementary figure: Comparison of jejunal and ileal starch digestibility

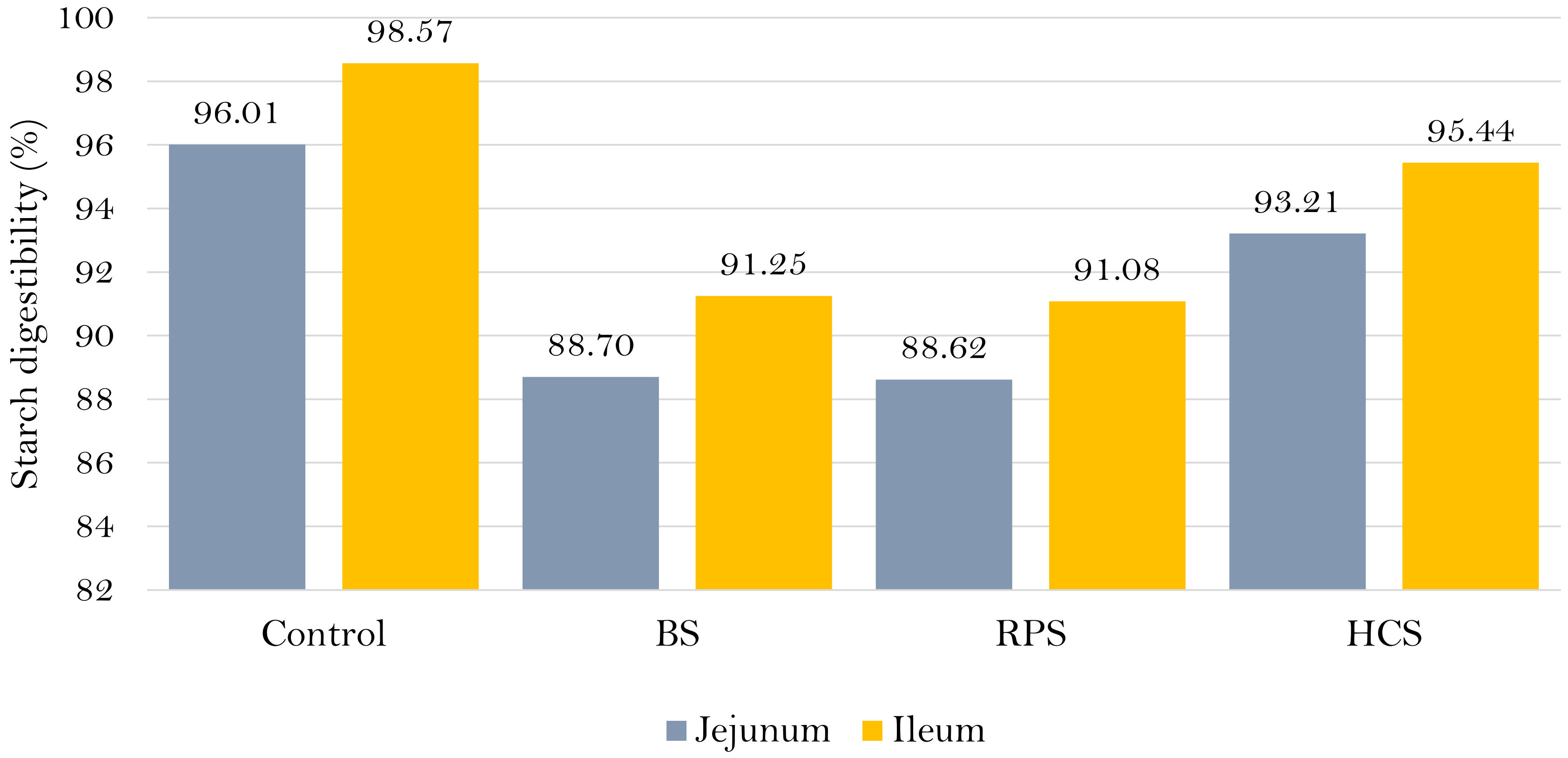

Note: The **marginal increase** in starch digestibility between jejunum and ileum was smaller for HCS than the other RS types

BS - Banana starch, RPS - Raw potato starch, HCS- High –amylose corn starch, RS - Resistant starch
